# Effectiveness and implementation success of a co-produced physical activity referral scheme in Germany: study protocol of a pragmatic cluster randomised trial

**DOI:** 10.1186/s12889-022-13833-2

**Published:** 2022-08-13

**Authors:** Anja Weissenfels, Sarah Klamroth, Johannes Carl, Inga Naber, Eriselda Mino, Wolfgang Geidl, Peter Gelius, Karim Abu-Omar, Klaus Pfeifer

**Affiliations:** Department of Sport Science and Sport, Friedrich-Alexander-Universität Erlangen-Nürnberg, Gebbertstraße 123b, 91058 Erlangen, Germany

**Keywords:** Physical activity, Physician-initiated PA promotion, Physical activity-related health competence, Cluster design, Pragmatic trial

## Abstract

**Background:**

While effective physical activity referral schemes (PARSs) and related structures for promoting physical activity (PA) already exist in several countries, in Germany, PARSs have not yet been implemented systematically and nationwide. Through a co-production approach with relevant actors in the German healthcare system, a PARS was developed, and an implementation plan was created (e.g. financing). This study protocol aims to evaluate the developed PARS for people with non-communicable diseases (NCDs) in Germany regarding its potential effectiveness and implementation success.

**Methods:**

To evaluate the effectiveness and implementation success of the PARS, we will apply a pragmatic cluster-randomised controlled trial (cRCT) in Hybrid II design by comparing two intervention groups (PARS vs PA advice [PAA]). The trial will take place in the Nürnberg metropolitan region, with 24 physician practices recruiting 567 people with NCDs. Both groups will receive brief PA advice from a physician to initially increase the participants’ motivation to change their activity level. Subsequently, the PARS group will be given individualised support from an exercise professional to increase their PA levels and be transferred to local exercise opportunities. In contrast, participants in the PAA group will receive only the brief PA advice as well as information and an overview of regional PA offerings to become more active at their own initiative. After 12 and 24 weeks, changes in moderate to vigorous PA and in physical activity-related health competence (movement competence, control competence, self-regulation competence) will be measured as primary outcomes. Secondary outcomes will include changes in quality of life. To measure implementation success, we refer to the RE-AIM framework and draw on patient documentation, interviews, focus groups and surveys of the participating actors (physicians, exercise professionals).

**Discussion:**

Through a between-group comparison, we will investigate whether additional individual support by an exercise professional compared to brief PA advice alone leads to higher PA levels in people with NCDs. The acceptance and feasibility of both interventions in routine care in the German healthcare system will also be evaluated.

**Trial registration:**

ClinicalTrials.gov, NCT04947787. Registered 01 June 2021.

**Supplementary Information:**

The online version contains supplementary material available at 10.1186/s12889-022-13833-2.

## Background

In Germany, only approximately 45% of adults are adequately physically active and meet the national and international physical activity (PA) recommendations of at least 150 min/week of moderate to vigorous activity or 75 min/week of intensive activity [1]. The worldwide increase in physical inactivity has led not only to an increase in non-communicable diseases (NCDs) and mortality rates but also to rising medical costs [2]. Regular PA has been associated with comprehensive positive physical and mental health effects, as scientifically demonstrated for more than 25 NCDs, including obesity, type 2 diabetes mellitus and cardiovascular diseases [3]. Despite these benefits, persons with chronic diseases in particular show considerably lower PA levels compared to healthy adults [4].

Physicians have been prescribing PA for over 2,500 years. The Indian physician Susruta, for example, recommended PA for his patients as early as 600 BC, and Hippocrates (460 – 370 BC) wrote the first-known exercise prescription for patients to manage chronic diseases [5]. Today, physician-initiated exercise is recommended in the European Union (EU) PA Strategy, the Global Action Plan on Physical Activity 2018–2030 (WHO) and German guidelines for PA and PA promotion (NEBB) as an effective PA measure [6, 7]. Accordingly, there are already established structures for physician-initiated PA promotion, such as ‘Exercise on referral’ (England) [8], ‘Physical activity on prescription’ (Sweden) [9] and ‘Green prescription’ (New Zealand) [10]. In Germany, physicians already make PA recommendations as part of routine care but without referring to an existing PA service or receiving financial compensation for the services provided [11].

The project *BewegtVersorgt*, funded by the German Federal Ministry of Health, aims to develop structures to promote PA for people with NCDs and to equip individuals with the competences necessary to lead a healthy, physically active lifestyle [12]. Based on a co-production process with relevant actors in the German healthcare system, a physical activity referral scheme (PARS) was developed, as detailed in Weissenfels et al. [13]. Following other promising referral schemes, key constituent elements, such as PA screening, assessment, counselling, referral form/prescription, feedback and follow-up [14], were identified and adapted to the structures of the German healthcare system. International studies have shown that these referral schemes have a meaningful impact, but the results vary widely, and merely transferring a specific concept to other healthcare systems is typically not feasible [15, 16].

Internationally, there are also simpler healthcare structures than PARS for PA promotion, such as PA advice (PAA), that provide good evidence for individuals’ initial motivation to change their PA behaviour but remain questionable regarding long-term PA promotion [17]. Therefore, the current study aims to compare a PARS and PAA intervention group to examine whether the individual support of an exercise professional using behaviour change techniques in the PARS group produces a more significant change in PA behaviour than in the PAA group.

### Objectives

Based on a Hybrid II design [18], the objectives of the pragmatic trial are twofold. The first objective is to evaluate the effectiveness of the developed PA interventions (PARS vs PAA) in terms of a) increasing moderate to vigorous PA, b) changes in physical activity-related health competence (PAHCO) (movement competence, control competence, self-regulation competence, see Carl et al. [19, 20]) and c) influencing the quality of life of people with NCDs. The second objective is (d) to test the success of the implementation plan. The following study protocol focuses particularly on evaluating effectiveness as more details on the success of the implementation plan will be presented in another article.

#### Primary hypotheses

The key elements of the PA interventions focus on the improvement of PAHCO and long-term changes in PA levels, so both outcomes are considered equally important as primary endpoints.

1. The PARS group members increases their (subjective) PA significantly after 12 and 24 weeks compared to the PAA group.

2) The PARS group members report significant improvements in PAHCO after 12 and 24 weeks compared to the PAA group.

#### Secondary hypothesis

3) The PARS group shows a significant increase in quality of life after 12 and 24 weeks compared to the PAA group.

#### Further hypothesis

4) Participating actors (physicians, exercise professions) are able to implement the developed PA interventions in routine care and evaluate them positively.

 

### Trial design

The study described in this protocol is a cluster-randomised controlled trial (cRCT) with two intervention arms (PARS vs PAA) and is designed to be pragmatic using the PRECIS-2 tool [21]. Based on the pragmatic application, the trial is guided by a Hybrid II design, following Curran et al. [18], which simultaneously tests the effectiveness of the interventions and the implementation plan. The study will be conducted as a pilot project in the Nürnberg metropolitan region, Germany, and will be scientifically supported by the Friedrich-Alexander-Universität Erlangen-Nürnberg (FAU), Germany.

**Fig. 1:**
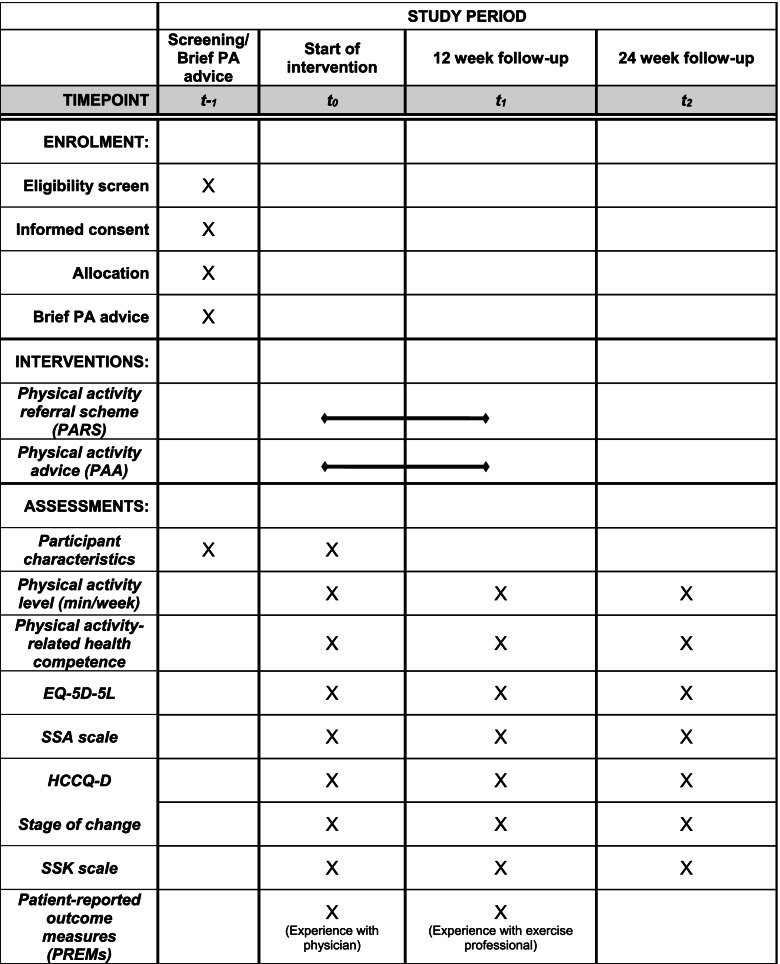
Schedule of enrolment, interventions, and assessments following SPIRIT. PA physical activity; PARS physical activity referral scheme; PAA physical activity advice; EQ-5D-5L European Quality of Life – 5 Dimensions – 5 Level; SSA self-efficacy towards physical activity; HCCQ-D Health Care Climate Questionnaire; SSK sport- and movement-related self-concordance; PREMs patient-reported outcome measures

This protocol is based on the Standard Protocol Items: Recommendations for Interventional Trials (SPIRIT) [22]. An overview of schedule of enrolment, both interventions, and assessments is provided in Fig. 1. The pragmatic trial will be conducted and reported in accordance with the reporting guidelines provided in the CONSORT 2010 statement [23], taking into account the CONSORT extension for pragmatic trials [24] and cRCTs [25].

## Methods

### Study setting and participants

The PARS was developed using a co-production approach that involved all relevant actors in the healthcare system (e.g. healthcare service providers, healthcare insurance providers, representatives of patient associations; *n* = 12 organisations). More information about the co-production process can be retrieved from the study protocol covering all project phases (development, implementation, evaluation, scaling-up) [13]. The pragmatic trial will take place in the German primary healthcare setting. As part of a routine examination, suitable patients will be recruited by physicians (general practitioners and medical specialists) and screened for eligibility. Participants are eligible for the study if they (a) are at least 18 years old and live in the Nürnberg metropolitan region; (b) have at least one of the following NCDs: controlled diabetes mellitus type 2, chronic cardiovascular disease, obesity (Body Mass Index [BMI] ≥ 30 kg/m^2^) or arthrosis in knee and/or hip; (c) do not meet official German PA recommendations (i.e. performing less than 150 min of moderate to vigorous aerobic activity per week); (d) are insured with health insurance companies cooperating with the project and (e) can safely participate in PAs based on medical judgement. Individuals will be excluded if they (a) plan to leave the Nürnberg metropolitan region during the study period, (b) are participating in another study, (c) plan to be absent for more than four weeks during the 12-week intervention, (d) have cognitive impairments that prevent an effective communication with the physician and the exercise professional, (e) have a mental illness, such as psychosis, substance abuse or mood and personality disorders, (f) have an unstable clinical situation or serious health impairments that prevent them from undertaking PA safely (e.g. acute myocardial infarction, unstable angina pectoris, fever, terminal tumour diseases).

### Recruitment procedures

#### Physicians

The recruitment of participants will take place via trained physicians in the Nürnberg metropolitan region. Participating general practitioners and medical specialists will be recruited through ‘quality circles’ (a continuing education programme for physicians), medical associations, a regional Bavarian science journal and the homepages of the project stakeholders. The project stakeholders consist of 12 organisations from the German healthcare system and include representatives from patients, the medical profession, the exercise professions, and insurance providers.

Inclusion criteria will be that the physicians (a) are located in the Nürnberg metropolitan region, (b) work in general medicine or specialise in internal medicine (cardiology, diabetology or endocrinology), orthopaedics, physical and rehabilitative medicine or geriatrics, (c) have expressed their willingness and commitment to participate in the study, (d) have adequate resources to manage the study and comply with the protocol, (e) have adequate patient volume according to participants’ inclusion criteria (given diseases, healthcare insurance) and (f) participate in a 90-min digital training session (introduction to motivational interviewing and interview guide for PA advice). Exclusion criteria will be (a) participation in conflicting studies and (b) practice relocation or retirement planned during the study period.

#### Participants

**Fig. 2:**
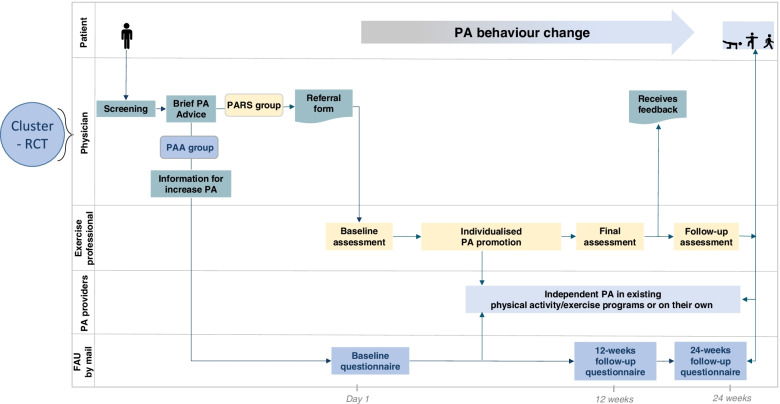
Study design with both groups (PARS vs PAA)

Suitable patients from the cooperating health insurance companies will be included in the study as part of their routine care (pragmatic trial). Eligible patients will be identified by the physicians or medical staff in advance (see inclusion and exclusion criteria above). Selected patients will receive information about the study and a screening assessment at their routine appointment (see Fig. 2), which will ask about their PA levels and contraindications. After a brief conversation about the benefits of PA with the physician, the patient can decide whether to participate in the programme and is referred to the next level of each intervention arm.

#### Exercise professionals (PARS group only)

Exercise professionals (e.g. physiotherapists, sport and exercise therapists) will be recruited using various strategies. An overview of all facilities related to PA promotion (physiotherapy practices, rehabilitation sports, prevention sports and fitness facilities related to health promotion) in the Nürnberg metropolitan region has already been compiled. The study team will systematically contact the responsible staff of each facility via telephone to present the study as well as general conditions and ascertain levels of interest. If the facility staff express interest, the responsible exercise professionals will participate in a 1.5-day digital training course in which they are familiarised with the intervention (individual PA promotion and assessments) and will have the opportunity to try out motivational interviewing. The study team will also advertise the project through project stakeholders’ websites, newsletters and information letters to the stakeholders’ members.

### Sample size

To calculate the number of participants, we focus on the primary endpoints of ‘moderate to vigorous PA (min/week)’ and ‘PAHCO’, including cluster adjustment. As the primary outcomes lead to four statistical tests, a Bonferroni correction is used accordingly with *α *= 0.05/4. With an assumed drop-out rate of 10%, a sample size of 567 participants from 24 medical practices (α = 0.0125, power = 80%) is required to detect differences between both intervention groups of low to moderate effect size (*f* = 0.175). To account for the effects of clustering, an intraclass correlation (ICC) of 0.02 is considered, estimated based on previous studies for cRCTs in primary care [26, 27]. We consider this ICC to be realistic in our trial as the participating practices are similarly structured (professional background, qualification level, treating patient group size and region) and were trained by the research team.

### Randomisation, allocation and blinding

A cRCT with randomisation at the physician practice level will be conducted for organisational reasons and to avoid the risk of mixing participants from both groups within a medical facility (contamination). The research team will recruit the physicians and provide them with detailed information about the study or answer questions. After physicians express interest in participating in the study and meet the inclusion criteria, they must provide written informed consent, which needs to be supported by the cooperating health insurance company. Each medical practice will be randomly allocated to the PARS or PAA group using computer-based block randomisation (Excel 2016). Due to the local density of physician practices in the Nürnberg metropolitan region, six blocks for randomisation will be defined (Nürnberg city [*n* = 10], Nürnberg surrounding area [*n* = 2], Fürth city [*n* = 10], Fürth surrounding area [*n* = 2], Erlangen city [*n* = 10] and Erlangen surrounding area [*n* = 2]). The practices will then be informed about further procedures, and physicians, as well as medical staff working in the facility, will undergo specific training according to the assigned intervention arms.

Because of group-specific procedures and the corresponding training, blinding the participating actors (physicians, exercise professionals) is not possible in this study. However, blinding participants is possible due to randomisation on the physician practices’ level. The participants will only be informed about the intervention in which they are involved and will not come into contact with the other intervention arm during the study. The statistician will also be blinded to the group allocation until completion of the statistical analysis.

### Interventions

The intervention period will last 24 weeks for the individual participant and will take place in the primary care setting of the German healthcare system. Both groups (PARS and PAA) will receive brief PA advice about increasing their activity levels. The study design is shown in Fig. 2. All trained actors in both groups will be asked to adhere to the intervention guidelines. For this, there are checklists to be completed and all participating actors as well as the participants are subsequently questioned about compliance. Participants are asked to maintain their lifestyle and not to start any other activities besides the study. Participants of both groups can quit the intervention at any time without giving any reason.

#### Physical activity referral scheme (PARS)

The PARS pathway is based on a conventional prescription for physical therapy in Germany and includes key actors, such as physicians, exercise professionals and exercise organisations. A detailed description of the pathway according to the PARS reporting checklist by Hanson et al. [28] can be found in Additional file 1.

The referral scheme begins with the physician’s screening. During a routine medical visit, the patient’s eligibility for participation in the study will be assessed. For this purpose, in addition to the inclusion and exclusion criteria described earlier, a short screening questionnaire will be used to determine average weekly PA. The questionnaire is based on three validated questionnaires for evaluating activity levels subjectively (Single-item [29], Physical Activity Vital Sign (PAVS) [30], Kaiser Permanente Southern California Exercise Vital Sign [31]) and is adapted to the trial setting. If the questionnaire reveals suitability – that is, the patient is inactive (< 150 min/week of moderate to vigorous PA) – brief PA advice (10 min) follows. The main goal is to initially motivate patients to change their behaviour and participate in the following intervention. The referral form can then be used to transfer the patient to a participating exercise professional.

After a 45-min initial assessment by the exercise professional, which is also part of the intervention, individually targeted PA promotion takes place over six sessions of 60 min each. The focus is on working out PA preferences with the patient, trying them and planning their subsequent implementation (e.g. in a regional exercise programme). For this purpose, exercise professionals can use a set of behavioural change techniques (BCTs), following Mitchie et al. [32], which has been identified as effective for PA behavioural change [33]. The BCTs are described in detail for both groups in Additional file 2. Exercise professionals in this study will focus particularly on the techniques of ‘goals and planning’, ‘feedback and monitoring’, ‘social support’, ‘shaping knowledge’ and ‘self-belief’. During the fourth session, a transfer to existing exercise or PA offers in the region will occur. Via a comprehensive brochure (more than 1000 offers from approximately 300 exercise providers), sorted by postal codes and types of offer, the participants will receive an overview of health-related PA opportunities and find the contact details for the respective exercise providers. After 12 weeks, a 45-min final assessment will take place to reflect on changes in the participant’s PA level and PAHCO and to formulate new goals for the next 12 weeks. The attending physician will receive feedback on the patient’s development. The transfer to existing PA programmes or independent activities will enable long-term maintenance of an active lifestyle, assessed in a follow-up assessment after a further 12 weeks. Before each assessment, participants will receive baseline or follow-up questionnaires to evaluate the effectiveness of the intervention and success of its implementation from their perspective. The questionnaires can be completed independently, although the treating exercise professional can assist with any questions.

#### Physical activity advice (PAA)

After being screened for eligibility, the PAA group will receive similar brief PA advicePAA to the PARS group. However, instead of being referred to exercise professionals for a more intensive intervention, the PAA participants will then receive a brochure with PA information, steps to start PA and adopt a more active lifestyle, specific information about the diseases targeted in this study and concrete tasks. The brochure also contains materials on BCTs, in which the BCTs ‘goals and planning’, ‘feedback and monitoring’ and ‘shaping knowledge’ are used but to a lesser extent than in the PARS group. The major difference between the two groups is the social support from an exercise professional, which is not available in the PAA group. An overview of the integrated BCTs for the PAA group is included in Additional file 2.

Like the PARS group, the PAA group will also receive the overview of local PA offers. A baseline questionnaire and follow-up questionnaire to be completed after 12 and 24 weeks will be sent by mail with stamped envelopes for their return to the project team. If the questionnaire has not been answered after 14 days, participants will receive a reminder by mail. If participants in this intervention arm have any questions about the brochure or completing the questionnaires, they can contact the research team by phone or email in specific time slots.

### Outcome measures

Except for participant characteristics, all data will be collected at the following three time points: after brief PA advice by the physician (basal), at 12 and at 24 weeks.

#### Participant characteristics

Participants’ characteristics will be captured through questions about age, gender, birthplace, marital status, social status, education level and income in the baseline questionnaire. Medical health status (type of disease) and medication intake will also be queried. Further specific medical characteristics prone to PA interventions, such as HbA1c, cannot be captured via questionnaires. For organisational reasons, these data cannot be retrieved systematically from all participating practitioners.

### Primary outcomes

#### Physical activity (PA) level

PA level will be measured by the Physical Activity, Exercise and Sport Questionnaire (Bewegungs- und Sportaktivität Fragebogen; BSA-F 3.0 [34]) – a validated German questionnaire that assesses the respondent’s amount of PA during the last four weeks. Based on the Frequency-Intensity-Time-and-Type (FITT) principles [35, 36], the questionnaire differentiates between leisure and transportation activities (PA score) and sport- and exercise-related activities (sport score). Participants report the frequency and duration of activities executed during the last four weeks. Minutes of leisure-time PA per week and sport-/exercise-related activity per week are calculated to gain the PA and exercise scores, which are combined to give the overall PA volume.

#### Physical activity (PA)-related health competence

Changes in PAHCO will be measured using the German version of the Physical Activity-Related Health Competence Questionnaire (BGK Questionnaire) [12, 37]. The questionnaire consists of 42 items that measure the following three sub-competencies necessary for health-promoting PA behaviour: movement competence (18 items; min = 0, max = 17.6), control competence (10 items; min = 0, max = 10.8) and self-regulation competence (14 items; min = 0, max = 14.8). Higher scores indicate higher competencies.

### Secondary outcomes

The European Quality of Life – 5 Dimensions – 5 Level (EQ-5D-5L) questionnaire is a self-administered quality of life scale that consists of a descriptive system and a visual analogue scale [38]. The descriptive system contains five well-being dimensions: mobility, self-care, usual activity, pain/discomfort and anxiety/depression, each of which has five levels of severity (no problems to incapacitated/extreme problem), coded from one to five. The result is a five-digit code that represents the participant’s health profile. The visual analogue scale assesses the participant’s overall current health.

In addition to the EQ-5D-5L results, the following secondary outcomes will be collected:

- Self-efficacy towards PA (SSA scale) [39]
- Participant’s perceived autonomy support (HCCQ-D) [40]
- Stage of change [41]
- Sport- and movement-related self-concordance (SSK scale) [42]

### Evaluation of the implementation plan

As well as effectiveness, the degree of implementation will be examined (see hypothesis 4) based on the five dimensions of the RE-AIM (Reach-Effectiveness-Adoption-Implementation-Maintenance) model [43], which include different aspects of implementation status and success at the individual and organisational levels. To examine the degree of implementation, a mixed method approach will be used, drawing on quantitative (e.g. characteristics of participating actors [Adoption] and patients reach [Reach]) and qualitative measurement procedures (e.g. adherence to guidelines [Implementation], adjustments during intervention [Implementation] and adoptions for long-term use [Maintenance]). For this purpose, a plan will be developed to capture various aspects in each dimension using different methods (e.g. interviews, focus groups, online surveys and document analysis) and at different moments during the intervention period. The perspectives of both the participating actors and patients will be addressed. Focusing on Patient-Reported Experience Measures (PREMs) [44], the patients’ perspectives will be retrieved with questionnaire surveys after 12 and 24 weeks, after which interviews or focus groups with patients may also be conducted. The actors will be asked for their perspectives during and after the interventions.

### Data collection and management

Within the pragmatic trial, participating actors (physicians and exercise professionals) will assist with data collection for the effectiveness evaluation in the PARS group by handing out the questionnaires. For the PAA group, the questionnaire will be delivered by mail. The questionnaire comprises a compilation of reliable and validated survey instruments (see ‘Outcome measures’ above) and will be completed independently by the study participants. To ensure standardised data collection, the participating actors will prepare for this task in a special training session. Due to the personal supervision of the PARS group participants, the data will be collected by the same exercise professional at all three measurement time points. In the PARS group, participants will receive the questionnaires prior to the baseline and at the 12- and 24-weeks follow-up assessments, while PAA group participants will receive them by mail and are expected to return them to the study team with a prepaid envelope. The study team will periodically collect the documents of the PARS group from the participating medical and therapeutic practices. Adverse events will be assessed in both groups via questionnaires at both 12 and 24 weeks. In the case of a study dropout, the exercise professional in the PARS group can also report specific reasons.

The data for evaluating the implementation plan will be collected by the study team during and after the intervention period. For example, data on the characteristics of the participating actors (e.g. physicians and exercise professionals) will be collected during the intervention, while information about the success of the interventions will only be collected after their completion. Planned interviews or focus groups will be conducted by trained researchers from the FAU study team.

All data will be transcribed, pseudonymised, recorded, archived and evaluated by the research team using quantitative methods (descriptive and inferential statistics via, e.g. SPSS, R) and qualitative social research methods (e.g. transcription and coding of structured interviews via MAXQDA). The data will be stored securely, and only direct project members of the FAU will have access to them. The deletion of all collected data is planned after the legal retention period of 10 years, beginning with the end of the project duration (30.11.2022).

### Data analysis

A linear modelling approach will be used to analyse the effectiveness of the intervention. The group information (PARS vs PAA) will constitute the decisive independent variable to be modelled on participants’ outcomes after the intervention. In this context, participants’ baseline values will be integrated into the calculations to control for potential differences before the intervention. Given the clustered design, it is necessary to account for the nested character of the data (level 2: physicians; level 1: patients). Depending on the final number of clusters included (level 2), participants recruited (level 1) and the power achieved, either physicians will be included as a control variable or the hierarchical structure will be considered through multilevel structural equation modelling (applying the Kenward-Roger correction for small cluster sizes [45]).

Adopting an intention-to-treat paradigm, we will include all participants who have been randomised in the analysis. To avoid underestimation of treatment effects through conservative replacement strategies (such as baseline observation carried forward), we will apply imputation techniques to this trial. Accordingly, dropouts and missing data will be treated by applying full information maximum likelihood (FIML) imputation. The Mardia test, with its inspection of statistical skewness and kurtosis, will determine multivariate normality. If the assumptions of normality are violated, the calculations will be based on robust maximum likelihood (MLR) procedures.

### Ethics and dissemination

The ethics application for *BewegtVersorgt* was submitted in written form to and approved by the responsible FAU institution (committee reference number 110_21 B). Any changes made to the study after the review will be reported immediately. The pragmatic trial adheres to the Declaration of Helsinki, and the data will only be published in pseudo-anonymised form to ensure that no assignment to persons can take place. Data protection rules will be respected throughout the project’s duration, and all participating actors and patients will be informed in advance about the study and required to give their written informed consent to participate. They will have the option of revoking this consent at any time without giving reasons. The study results will be published in peer-reviewed journals and presented at national and international congresses. Regarding dissemination and scaling-up strategies, the transfer and scaling-up of the referral scheme (national level, other target regions and groups) will be discussed in a working group of national experts. However, as the planning of the scaling-up process depends on the results of the effectiveness analysis and the implementation success, it can only be concretised at a later stage in the project.

## Discussion

The study aims to improve PA, PAHCO and quality of life among inactive people with NCDs via physician-initiated PA promotion. It will also examine whether the implementation of such PA interventions, especially the referral scheme, is successful in the German healthcare system or whether adjustments have to be made.

We expect that individuals in the PARS group, who receive PA support from exercise professionals, will show significantly higher increases in their PA levels and PAHCO than those in the PAA group. We will also evaluate the participants’ adherence to selected activities at the 24-week follow-up assessment. The largest difference between the interventions that may influence individual behaviour change is the extent to which BCTs, and the ‘social support’ category in particular, are used. If the PAA group participants also succeed in making positive changes, it will be necessary to discuss which intervention can be used most efficiently to produce the desired health effects.

The execution of the comprehensive evaluation concept in this pragmatic trial is largely dependent on the recruitment rate in the physicians’ practices and the implementation success of the PA interventions in routine care. Thus, an assessment of the results and an adjustment of the evaluation concept will only be possible once the pragmatic study is in progress. While we also acknowledge that a longer follow-up period would be desirable, it is not possible due to the funding amount and period of the project (including the co-production approach and planning).

Through the results and process of co-productive development with stakeholders in the German healthcare system, we hope to facilitate a potential transfer to the national level and to other target regions or groups. As a result of the co-production process, the intervention’s adaptation to the legal and financial conditions of the healthcare system has already laid a foundation for such a transfer and may ease the subsequent transfer to routine care.

In summary, the results of this study will make it possible to evaluate the PA-promoting effects of PARS in primary healthcare in Germany, laying the foundation for nationwide dissemination/scaling up.

### Trial status

NCT04947787 (Status: Recruiting).

## Supplementary Information

**Additional file 1.** Supplementary material_PARS Checklist.Physical Activity Referral Scheme (PARS) Reporting Checklist.

**Additional file 2.** Supplementary material_BCT. Overview of the BCTs used in the interventions according to Michie et al. [32].

## Data Availability

Data will be available upon reasonable request from Dr Anja Weissenfels; Email: anja.weissenfels@fau.de.

## Abbreviations

CONSORT: Consolidated Standards of Reporting Trials
BCT: Behaviour change techniques
BMI: Body mass index
EQ-5D-5L: European Quality of Life – 5 Dimensions – 5 Level
HCCQ-D: Health Care Climate Questionnaire
ICC: Intraclass correlation
NEBB: German guidelines for PA and PA promotion
PAHCO: Physical activity-related health competence
PAVS: Physical Activity Vital Sign
SSA: Self-efficacy towards physical activity
SSK: Sport- and movement-related self-concordance
cRCT: Cluster-randomised controlled trial
FAU: Friedrich-Alexander Universität Erlangen-Nürnberg
FIML: Full information maximum likelihood
FITT: Frequency-Intensity-Time-and-Type
MLR: Robust maximum likelihood
NCD: Non-communicable disease
PA: Physical activity
PAA: Physical activity advice
PARS: Physical activity referral scheme
PREM: Patient-reported experience measure
SPIRIT: Standard Protocol Items: Recommendations for Interventional Trials
WHO: World Health Organization
