# Supplementary material for: Effectiveness and implementation success of a co-produced physical activity referral scheme in Germany: study protocol of a pragmatic cluster randomised trial
Source: BMC Public Health. 2022 Aug 13;22:1545. doi: 10.1186/s12889-022-13833-2 (PMC9375362; doi:10.1186/s12889-022-13833-2)
Supplement: Supplementary file 1 — Additional file 1. Supplementary material_PARS Checklist.Physical Activity Referral Scheme (PARS) Reporting Checklist. [file 12889_2022_13833_MOESM1_ESM.pdf]

**Physical Activity Referral Scheme (PARS) Reporting Checklist**		
**Level 1 PARS classification**		
**Level 1a: Primary classification**<br><br>The purpose of this taxonomy is to provide a classification system for PARS, including clinically based exercise schemes, exercise referral schemes and social prescribing for physical activity (PA). It is for use in evidence reviews of delivery and effectiveness. It is also an audit and monitoring tool for funders and providers to capture service delivery. The taxonomy is intended for programmes that fulfil all of the following **three criteria**:		Tick all that apply
1. Have a primary aim of increasing PA		x
2. Have a formalised referral process		x
3. Are for individuals who are inactive and/or sedentary, and/or have (*or are at risk of having)* a health condition.		x
**If you have not ticked all of these boxes, then the PARS taxonomy is not suitable for your programme.**		
Additionally programmes **may** also include the following		Tick any that apply
1. Individual behaviour change consultations *(explicit, planned behaviour change techniques included e.g. goal setting, formalised activity tracking/activity monitoring)*		x
2. PARS specialist staff supervised PA sessions or one-to-one supervision		x
3. Signposting to a range of generic available activities delivered by non-PARS specialist staff *(e.g. walking football, yoga, Pilates, Zumba)*		x
**1b Provider**	Tick all that apply	Further specify provider
Leisure trust		
Local government		

Other third/community sector organization		
Sport-based (governing bodies or sport clubs)		
Commercial/private provider		
Health (e.g. NHS)	x	Two health insurance providers
Other (define)		
**1b Setting**	Tick all that apply	Specify exact location (e.g. leisure centre name and location, including postcode or web link/app name etc.)
Leisure centre		
Green/outdoor space (define)		
Sports club		
Community facility (define)		
Commercial gym		
Other commercial facility (define)		
Other local government facility (define)		
Home-based		
Clinical setting	x	Physician and physiotherapy practices; Names and locations can be found here: https://www.bewegtversorgt.fau.de/teilnehmen/
Online/eHealth/mHealth		
Other (define)		
**1c Conditions accepted (have or at risk of)**	Tick all that apply	Specify exact conditions within each subsection
Cardiovascular primary prevention (e.g. hypertension)	x	Hypertension
Cardiovascular secondary prevention (e.g. acute coronary syndrome, heart failure, stroke)	x	Chronic cardiovascular disease (chronic ischaemic heart disease, cardiomyopathy, heart failure)
Respiratory disease (e.g. chronic obstructive pulmonary disease, asthma)		
Metabolic disease (e.g. type 2 diabetes)	x	Type 2 diabetes

Mental health condition/disability (e.g. anxiety, depression, schizophrenia)		
Learning disability (e.g. autism spectrum disorder)		
Musculoskeletal (e.g. back pain, osteoarthritis)	x	Arthrosis of knee or hip
Cancer (nonspecific)		
Cancer specific (e.g. breast, bowel)		
Weight loss or weight maintenance	x	Obesity
Falls prevention (primary and secondary prevention)		
Neurodegenerative disease (dementia, Alzheimer's Parkinson's)		
Inactive and/or sedentary		
Other (define)		
**1d Activity type**	Tick all that apply	Further specify activities
Gym-based (cardiovascular and/or strength)		
PARS specialized class led by PARS qualified staff (e.g. U.K level 3 exercise referral qualification)		
Walking		
Jogging/running		
Swimming		
Outdoor cycling, e-bikes		
Sport (e.g. badminton, walking football)		
Seated fitness class		
Generic fitness class (e.g. yoga, aerobics, Zumba)		
Gardening/green gym or other green health activity		

PA education sessions	x	Six 1-hour sessions of individual PA promotion
Other (define)	x	Highly individualized; PA activities will be chosen based on participant preferences and underlying health status. They can vary from self-directed activities such as walking to organized group-based classes offered by the local PA providers.
**1e Funding**	Tick all that apply	Please state exact funding source, level of funding per participant and length of funding agreement
Fully externally funded	x	The PARS is funded by health insurance providers for 24 weeks.
Partially externally funded		
Fully internally funded (e.g. core organization budget)		
Partially internally funded		
Participants pay for PARS		
Other (define)		
**Level 2 PARS Characteristics**		
**2a Staff structure**	Tick all that apply	Define
Contracted staff		
Self-employed	x	Physicians and exercise professionals in private practices
Volunteers		
Other (define)	x	Routine care staff
**2b Staff qualifications**	Tick all that apply	Define exact qualifications held
PARS qualification	x	Physicians will have a 90-minute digital training including an introduction to motivational interviewing and the delivery of the PA advice. The practice staff will be trained regarding to the organisational aspects of PARS. The exercise professionals will receive a 1.5-day digital training course in which they learn about the intervention content (individual PA promotion, assessments) and try out practical applications of motivational interviewing.
Condition specific qualification		

Other (state)		
**2c To the best of your knowledge, is the scheme based on one or more behaviour change theories?**		Please state if you know what theory your scheme is based on
Yes	x	Physical Activity-Related Health Competence model (PAHCO)
No		
**2d To the best of your knowledge, does the scheme use one or more behaviour change techniques?**		Please state if you know what techniques your scheme uses
Yes	x	Goals and planning, feedback and monitoring, social support, shaping knowledge, natural consequences, comparison of behaviour, associations, repetition and substitution, comparison of outcomes, regulation, antecedents, identity, self-belief
No		
**2e Referral Source**	Tick all that apply	No. of referrals per year
Primary care	x	Not yet available
Secondary care		
Tertiary care		
Self-referral		
Other (state)		
**2f Referrers**	Tick all that apply	Additional comments
General practitioner	x	Physicians of general medicine or specialists in internal medicine (cardiology, diabetology or endocrinology), orthopedics, physical and rehabilitative medicine, or geriatrics
Practice nurse		
Rehabilitation professional (state profession)		
Self-referral		
Social prescriber (e.g. link worker/health trainer)		

**2g Referral process**	Tick all that apply	Additional comments
Email		
Printed and mailed to participant		
Printed and given to participant to take to PARS	x	The participants receives a contact list of participating exercise professionals and should call for an appointment
Via online portal		
Other (define)		
**2h Scheme duration**	Tick one	State exact duration
Number of weeks client can attend scheme	x	24 weeks
Total number of sessions		
No defined length (open-ended)		
Other		
**2i Session frequency**	State	
Number of sessions per participant, per week	n/a	The scheme involves six sessions scheduled throughout the first 12 weeks after the baseline assessment. Final and Follow-up assessments are scheduled at week 12 and 24 respectively.
**2j Session length**	State	
Define session length	30-60 min	Baseline and Final assessment: 45 minutes<br>Individual PA promotion sessions: 60 minutes<br>Follow-up assessment: 30 minutes
**2k Session time**	Tick all that apply	Define time span of available sessions (e.g. 10.00-12.00pm)
Morning		
Afternoon		
Evening		
Weekday		
Weekend		

**2l Physical activity session type**	Tick all that apply	Further define session type (e.g. PARS supervised circuit session or independent walking football option)
PARS-supervised group-based sessions		
PARS-supervised individual sessions		
Independent PA following assessment	x	Independent PA in existing PA/exercise programs or on their own.
Generic PARS-supervised sessions for all conditions		
Condition specific PARS-supervised sessions		
Independent PA choices without assessment		
PA education sessions		
Technology-based support *(e.g. mHealth app or web-based)*		
Other (define)		
**2m Exit routes**	Tick all that apply	Give details of exit routes activities
Formal exit route (defined sessions for completers)		
Signposting to other activities	x	During the fourth individualized PA promotion session, a transfer to existing exercise or PA offers in the region occurs. Via a comprehensive brochure (more than 1000 offers from approx. 300 exercise providers), sorted by postal codes and types of offer, the participants receive an overview of health-related activity opportunities and find contact details for the respective PA/exercise providers.
Open-ended (no exit route required)		
None (state why no exit route provided)		
**2n Action in case of non-attendance** *Is there a standardised procedure for non-attendance?*	Tick all that apply	Specify time points, number of attempts to contact and by whom

**Yes**	x	The contact method depends on the facility.
Participant contacted by letter		
Participant contacted by technology-based support		
Participant contacted by telephone		
Participant contacted by text		
Participant not contacted		
Other (define)		
**No**		
**2o Baseline assessment**	Tick one	State when this occurs (e.g. prior to first PA session or at first PA session)
Yes	x	Prior to the first individualized PA promotion session
No		
**2p Exit assessment**	Tick one	State when this occurs (e.g. after 12 weeks, or after 24 sessions)
Yes	x	12 and 24 weeks after the baseline assessment
No		
**2q Feedback provided to referrer**	Tick all that apply	State what is included and how feedback is provided (e.g. attendance and via email).
**Yes** (state what is included)	x	Change in everyday and leisure activities, motivational status, particularities that might happen during the intervention delivery, and when the attendance was stopped. The feedback sheet will be send by mail.
How is feedback provided? (state)		
**No**		
**2r Exclusion criteria**	Tick all that apply	State specific exclusion criteria
**Yes**	x	- planning to leave the Nürnberg metropolitan region during the study period<br>- taking part in another study<br>- planning to be absent for more than four weeks during the next three month<br>- having cognitive impairments that prevent an effective communication with the PARS staff<br>- having mental illness such as psychotic, substance abuse, mood, personality disorders

		- having any disease or unstable clinical situation that prevent from undertaking physical activity safely (e.g. pain in the chest when performing physical activity, loss of balance because of dizziness or loss of consciousness, etc.)
**No**		
**Level 3 Participant Measures**		
**3a Demographics**		
3a.1 Sex	Tick if recorded	Define
State categories	x	Male, female, divers
3a.2 Age at the point of referral	Tick if recorded	Define
Individual age recorded	x	
Minimum age	x	18
Maximum age		
3a.3 Socio-economic status	Tick if recorded	Define
Postcode/zipcode recorded	x	Street, zip code, city
3a.4 Ethnicity	Tick if recorded	Define
State ethnic categories		
3a.5 Employment status	Tick if recorded	Define
State employment categories	x	Fully employed, part-time employed, unemployed (pension, full-time college, or unemployed)
3a.6 Education status	Tick if recorded	Define
State education categories	x	Graduation after a maximum of 7 years of school attendance; secondary//elementary school; middle school; high school, general of subject-linked higher education entrance qualification, advanced technical college entrance qualification, technical secondary school; other qualification.
3a.7 Other demographic measure	Tick if recorded	Define

State what other measures and how they are defined	x	Occupation (employed person, civil servant, farmer, self-employed with or without employees, apprentice/intern/volunteer, voluntary social/ecological/cultural year, never been employed before)<br>Net household income (open, categories)
**3b Monitoring and evaluation**		
3b.1 Number of referrals	Tick if recorded	Additional comments
Number of referrals received per annum	n/a	The number of referrals will be reported when the study is concluded.
3b.2 Uptake, attendance and adherence (please identify the definitions used)	Tick if recorded	Define measures
Uptake of intervention *(number of referrals who attend baseline assessment)*	x	Number of persons that were offered participation in the PARS and accept to receive brief PA advice.
Uptake of PA *(number of referrals that attend at least one PA session)*	x	Uptake of the individualized PA intervention: number of referrals that attend the baseline assessment.
Adherence to intervention *(number of referrals that attend exit assessment)*	x	Number of referrals that attend the follow-up assessment.
Adherence to PA *(number of referrals that attend an agreed number of sessions e.g. 60% of programme contact time)*	n/a	
Attendance *(number of attendances in a defined period)*	x	Number of sessions attended out of nine sessions (six individualized PA promotion sessions, three assessment sessions).
**3c Measures of change**	Tick if recorded	Define time points (e.g. baseline and week 24).
Change in PA behaviour (define measure)	x	Baseline, 12 and 24 weeks<br>Physical Activity, Exercise, and Sport Questionnaire (Bewegungs- und Sportaktivität Fragebogen; BSA-F 3.0)
Change in wellbeing (define measure)	x	Baseline, 12 and 24 weeks<br>Quality of life (European Quality of Life – 5 Dimensions – 5 Levels Questionnaire; EQ-5D-5L)
Change in physiological measures (e.g. BP, weight, % weight change, BMI)		
Other (define)	x	Baseline, 12 and 24 weeks:

		- Physical activity-related health competence (PAHCO)<br>- Self-efficacy towards physical activity (SSA scale)<br>- Participant’s perceived autonomy support (HCCQ-D)<br>- Stage of change<br>- Sport- and movement-related self-concordance (SSK scale)
