# Supplementary material for: Effectiveness and implementation success of a co-produced physical activity referral scheme in Germany: study protocol of a pragmatic cluster randomised trial
Source: BMC Public Health. 2022 Aug 13;22:1545. doi: 10.1186/s12889-022-13833-2 (PMC9375362; doi:10.1186/s12889-022-13833-2)
Supplement: Supplementary file 2 — Additional file 2. Supplementary material_BCT. Overview of the BCTs used in the interventions according to Michie et al. [32]. [file 12889_2022_13833_MOESM2_ESM.pdf]

Behaviour Change Techniques	Physical Activity Referral Scheme – Identification and Description		Physical Activity Advice – Identification and Description	
1 Goals and planning	√	**Content**: goal setting (behaviour) [1.1], problem solving [1.2], goal setting (outcome) [1.3], action planning [1.4], goal setting [1.5], review behaviour goals [1.6], discrepancy between current behaviour and goal, review outcome goal(s) [1.7], commitment [1.9]<br>**Materials:** PA pyramid (information sheet), PA goal setting (worksheet), barrier management for PA (worksheet), PA diary (worksheet), PA action plan (worksheet), finding PA offers (worksheet), regional PA offers (brochure)<br>**Execution:** guided by an exercise professional during session 1, sessions 4-6, follow-up assessment	√	**Content**: goal setting (behaviour) [1.1], problem solving [1.2], goal setting (outcome) [1.3], action planning [1.4], goal setting [1.5], review behaviour goals [1.6], discrepancy between current behaviour and goal, review outcome goal(s) [1.7]<br>**Materials:** PA pyramid (information sheet), PA goal setting (worksheet), barrier management for PA (worksheet), PA diary (worksheet), PA action plan (worksheet), regional PA offers (brochure)<br>**Execution:** conducted independently with the help of two brochures
2. Feedback and monitoring	√	**Content:** feedback on behaviour [2.2], self-monitoring of behaviour [2.3], self-monitoring of outcome(s) of behaviour [2.4], feedback on outcome(s) of behaviour [2.7]<br>**Materials:** clinical report**,** PA diary (worksheet), mood assessment (worksheet), PA-related health benefits (information sheet), PA benefits (worksheet), PA goal setting (worksheet)<br>**Execution:** guided by an exercise professional during baseline assessment, sessions 1-6, final assessment, follow-up assessment	√	**Content:** self-monitoring of behaviour [2.3]<br>**Materials:** mood assessment (worksheet), PA-related health benefits (information sheet), PA-related health benefits (information sheet), PA benefits (worksheet), PA goal setting (worksheet)<br>**Execution:** conducted independently with the help of a brochure
3. Social support	√	**Content:** social support (unspecified) [3.1], social support (practical) [3.2], social support (emotional) [3.3]<br>**Materials:** -<br>**Execution:** guided by a physician during the brief PA advice and by an exercise professional during baseline assessment, sessions 1-6, final assessment, follow-up assessment	√	**Content:** social support (unspecified) [3.1]<br>**Materials:** -<br>**Execution:** guided by a physician during the brief PA advice
4. Shaping knowledge	√	**Content:** instruction on how to perform a behaviour [4.1], re-attribution [4.3]<br>**Materials:** PA diary (worksheet), PA pyramid (information sheet), PA preferences (worksheet), PA cards (worksheet), PA-related health benefits (information sheet), disease-specific PA advices (information sheet), intensity control with Borg Scale (worksheet), defining optimal heart rate for PA (worksheet), defining optimal PA intensity (information sheet), mood assessment (worksheet), finding PA offers (worksheet), regional PA offers (brochure), PA goal setting (worksheet), PA action plan (worksheet), PA benefits (worksheet), supportive factors (worksheet), barrier management for PA (worksheet), strategies to overcome barriers to PA (information sheet), examples of endurance/strength training intensity progression (information sheet)<br>**Execution:** guided by an exercise professional during baseline assessment, sessions 1-6	√	**Content:** instruction on how to perform a behaviour [4.1], re-attribution [4.3]<br>**Materials:** PA diary (worksheet), PA pyramid (information sheet), PA preferences (worksheet), PA cards (worksheet), PA-related health benefits (information sheet), disease-specific PA advices (information sheet), intensity control with Borg Scale (worksheet), defining optimal heart rate for PA (worksheet), mood assessment (worksheet), regional PA offers (brochure), PA goal setting (worksheet), PA action plan (worksheet), PA benefits (worksheet), barrier management for PA (worksheet), strategies to overcome barriers to PA (information sheet)<br>**Execution:** conducted independently with the help of two brochures

5. Natural consequences	√	**Content:** information about health consequences [5.1], monitoring of emotional consequence [5.4]<br>**Materials:** mood assessment (worksheet), PA-related health benefits (information sheet), PA benefits (worksheet), clinical report<br>**Execution:** guided by an exercise professional during sessions 1-6, final assessment, follow-up assessment	√	**Content:** information about health consequences [5.1], monitoring of emotional consequence [5.4]<br>**Materials:** mood assessment (worksheet), PA-related health benefits (information sheet), PA benefits (worksheet)<br>**Execution:** conducted independently with the help of a brochure
6. Comparison of behaviour	−	**Content:** -<br>**Materials:** -<br>**Execution:** -	−	**Content:** -<br>**Materials:** -<br>**Execution:** -
7. Associations	√	**Content:** exposure [7.7]<br>**Materials:** PA diary (worksheet), PA goal setting (worksheet)<br>**Execution:** guided by an exercise professional during session 1, sessions 5-6, final assessment, follow-up assessment	√	**Content:** exposure [7.7]<br>**Materials:** PA diary (worksheet), PA goal setting (worksheet)<br>**Execution:** conducted independently with the help of a brochure
8. Repetition and substitution	√	**Content:** behavioural practice/rehearsal [8.1], behaviour substitution [8.2], habit formation [8.3], habit reversal [8.4], generalization of a target behaviour [8.6], graded tasks [8.7]<br>**Materials:** PA diary (worksheet), PA goal setting (worksheet), intensity control with Borg Scale (worksheet), defining optimal heart rate for PA (worksheet), defining optimal PA intensity (information sheet), mood assessment (worksheet), clinical report<br>**Execution:** guided by an exercise professional during sessions 2-6, final assessment, follow-up assessment	√	**Content:** behavioural practice/rehearsal [8.1], behaviour substitution [8.2], habit formation [8.3], habit reversal [8.4], generalization of a target behaviour [8.6], graded tasks [8.7]<br>**Materials:** PA diary (worksheet), PA goal setting (worksheet), intensity control with Borg Scale (worksheet), defining optimal heart rate for PA (worksheet), defining optimal PA intensity (information sheet), mood assessment (worksheet)<br>**Execution:** conducted independently with the help of a brochure
9. Comparison of outcomes	√	**Content:** pros and cons [9.2]<br>**Materials:** PA goal setting (worksheet), PA-related health benefits (information sheet), PA benefits (worksheet), mood assessment (worksheet)<br>**Execution:** guided by an exercise professional during session 1, sessions 5-6	√	**Content:** pros and cons [9.2]<br>**Materials:** PA goal setting (worksheet), PA-related health benefits (information sheet), PA benefits (worksheet), mood assessment (worksheet)<br>**Execution:** conducted independently with the help of a brochure
10. Reward and threat	√	**Content:** Social reward [10.4]<br>**Materials:** -<br>**Execution:** guided by an exercise professional during sessions 1-3, sessions 5-6, final assessment, follow-up assessment	−	**Content:** -<br>**Materials:** -<br>**Execution:** -
11. Regulation	√	**Content:** reduce negative emotions [11.2]<br>**Materials:** -<br>**Execution:** guided by an exercise professional during sessions 2-3, sessions 5-6	−	**Content:** -<br>**Materials:** -<br>**Execution:** -
12. Antecedents	√	**Content:** body changes [12.6]<br>**Materials:** clinical report<br>**Execution:** guided by an exercise professional during baseline assessment, final assessment, follow-up assessment	−	**Content:** -<br>**Materials:** -<br>**Execution:** -

13. Identity	√	**Content:** incompatible beliefs [13.3], valued self-identity [13.4]<br>**Materials:** PA preferences (worksheet), PA cards (worksheet), barrier management for PA (worksheet), supportive factors (worksheet), PA diary (worksheet), PA benefits (worksheet), strategies to overcome barriers to PA (information sheet)<br>**Execution:** guided by an exercise professional during sessions 1-6, final assessment, follow-up assessment	√	**Content:** incompatible beliefs [13.3], valued self-identity [13.4]<br>**Materials:** PA preferences (worksheet), PA cards (worksheet), PA diary (worksheet), barrier management for PA (worksheet), PA benefits (worksheet), strategies to overcome barriers to PA (information sheet)<br>**Execution:** conducted independently with the help of a brochure
14. Scheduled consequences	−	**Content:** -<br>**Materials:** -<br>**Execution:** -	−	**Content:** -<br>**Materials:** -<br>**Execution:** -
15. Self-belief	√	**Content:** verbal persuasion about capability [15.1], focus on past success [15.3]<br>**Materials:** clinical report, PA diary (worksheet), PA goal setting (worksheet)<br>**Execution:** guided by an exercise professional during baseline assessment, sessions 1-6, final assessment, follow-up assessment	−	**Content:** -<br>**Materials:** -<br>**Execution:** -
16. Covert learning	−	**Content:** -<br>**Materials:** -<br>**Execution:** -	−	**Content:** -<br>**Materials:** -<br>**Execution:** -

[ ] number of Behaviour Change Techniques subcategory

PA physical activity
